# Microwave-Assisted Enzymatic Extraction of Flavonoids from *Armeniaca mume* Sieb. Blossom and Their Immunomodulating Effect in Mice with DSS-Induced Colitis

**DOI:** 10.3390/molecules26040855

**Published:** 2021-02-06

**Authors:** Xinjun Yao, Jicheng Xu, Benu Adhikari, Weiqiao Lv

**Affiliations:** 1 College of Biological and Food Engineering, Anhui Polytechnic University, Wuhu 241000, China; yxj1969416508@163.com; 2 School of Science, RMIT University, Melbourne, VIC 3083, Australia; 3 College of Engineering, China Agricultural University, Beijing 100083, China; lvweiqiao@cau.edu.cn

**Keywords:** microwave-assisted, enzymatic extraction, *A. mume* Sieb. blossom, immune regulation

## Abstract

*Armeniaca mume* Sieb. blossom is among the traditional Chinese edible flowers, and it is widely used in the food and pharmaceutical industries. Flavonoids are among the most abundant bioactive compounds in *A. mume* Sieb. blossom. However, the research on the extraction of flavonoids from *A. mume* Sieb. blossom and their immunomodulating function is insufficient. In this study, we developed a microwave-assisted enzymatic extraction of flavonoids from *A. mume* Sieb. blossom (FAMB) and explored their immunomodulating effect on mice with dextran sulfate sodium salt-induced colitis. The results showed that the optimum parameters for microwave-assisted enzymatic extraction of FAMB were as follows: cellulase: 2.0%; microwave power: 200 W; microwave action time: 5 min; and enzymatic hydrolysis time: 50 min. FAMB significantly promoted the lymphocyte proliferation and natural killer (NK) cell killing activity in colitis mice, and increased the concentrations of TNF-α, IFN-γ, and IL-2 in serum. FAMB also significantly reduced the apoptosis of spleen lymphocytes in these mice. These results demonstrated that the microwave-assisted enzymatic method could significantly improve the yield and efficacy extraction of FAMB. FAMB showed a good immunomodulation effect on colitis mice.

## 1. Introduction

The blossom of *Armeniaca mume* Sieb. is among the traditional Chinese edible flowers and is widely used in the food and drug industries [1]. It has been proven by pharmacological and clinical trials that *A. mume* Sieb. blossom has a broad spectrum of benefits including antiviral, antitumor, immune-enhancing, and other pharmacological effects [2,3]. The extracts of *A. mume* Sieb. blossom have been shown to possess a strong antibacterial effect against a variety of bacteria [4]. Flavonoids are among the main active components in *A. mume* Sieb. blossom and have significant antioxidant effects. These flavonoids have been shown to have antitumor, hypoglycemia, lipidemia, liver protection, and gall bladder protection effects through clinical trials [5,6,7]. The uniqueness of these flavonoids is used as one of the criteria for evaluating the quality of *A. mume* Sieb. blossom.

The extraction methods of flavonoids used in Chinese medicine primarily include solvent extraction, ultrasonic extraction, and enzymatic hydrolysis [8,9,10]. Each extraction method has its advantages and disadvantages. Their disadvantages are generally manifested in low raw material utilization, long production cycle, and low extraction efficiency [11,12,13]. Microwave-assisted enzymatic extraction is a new method, as targeted and rapid heating of water molecules facilitates the separation of total flavonoids from the cell walls of prunes. Enzymatic extraction also comes with the advantages of mild processing conditions, low pollution, and less safety risk. Enzymes are able to break down the plant cell walls to a limited extent. However, microwave-assisted extraction dissolves cell walls, quickly enabling more effective extraction of total flavonoids [14,15,16]. Currently, most research is focused on fruits of *A. mume* Sieb., but not much on its blossom. The antioxidant and immune boosting functions of flavonoids of *A. mume* Sieb. blossom (FAMB) are still unclear. 

The objective of this study was to improve the extraction efficacy or yield of flavonoids from *A. mume* Sieb. blossom, and then further determine the effect of these flavonoids on lymphocyte proliferation; natural killer (NK) cell killing activity; TNF-α, IFN-γ, and IL-2 concentrations in serum; and the spleen lymphocyte apoptosis of colitis mice. *A. mume* Sieb. blossom was used as raw material, and a microwave-assisted enzymatic method was used to extract FAMB. The results show that FAMB exerts an immunomodulatory effect on mice with colitis, and lay the foundation for further research on biological activity of FAMB and other functional compounds found in *A. mume* Sieb. blossom.

## 2. Materials and Methods

### 2.1. Raw Materials

*A. mume* Sieb. blossom was picked at the *A. mume* Sieb. planting base of Liuliumei Co., LTD in Wuhu, China. Acidic cellulase (200,000 U/g) was purchased from Anhui Yinqiao Biotechnology Co., LTD. Clean grade male mice (8–12 weeks old) were purchased from Changzhou Cavins Experimental Animals Co., LTD. The laboratory animal procedures were conducted in strict accordance with the National Institutes of Health Guidelines. The experimental protocol was approved by the Animal Ethics Committee of Anhui Polytechnic University. Fetal bovine serum (FBS) and RPMI-1640 medium were purchased from Seymour Fly Bio-Chemical Products Co., LTD. Cyclophosphamide (CTX), AGAR medium, 3-(4,5-dimethylthiazole-2)-2,5-diphenyltetrazolium bromide (MTT), dimethyl sulfoxide (DMSO), and enzyme-linked immunosorbent assay (ELISA) kits were purchased from Bomer Biotechnology Co., LTD. Yac-1 and P815 cells were provided by the Chinese Academy of Sciences. All other reagents were of analytical grade purity.

### 2.2. Extraction of FAMB

**Figure 1:**
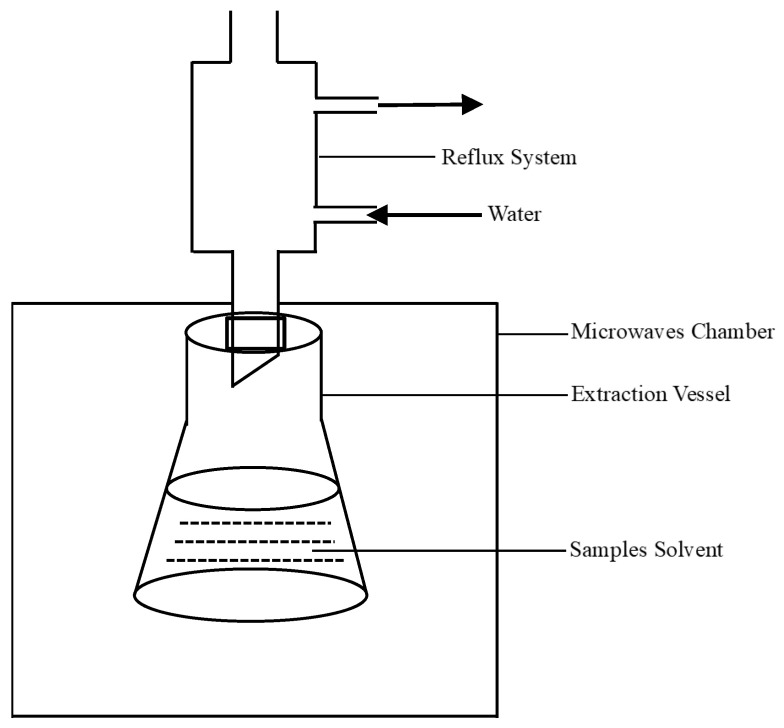
The schematic diagram of the microwave-assisted enzymatic extraction of flavonoids.

*A. mume* Sieb. blossom was dried and ground. Powder (10.0 g) was accurately weighed in each run, and 300 mL distilled water was added. Cellulase of different mass percentages, different microwave power levels, different microwave time, and different enzymatic hydrolysis time was used to extract flavonoids. The experimental procedure was as follows: dried *A. mume* Sieb. blossom → grinding → microwave-assisted enzymatic extraction → centrifugation (955× *g*) for 20 min → determination of flavonoid content by aluminum nitrate colorimetric method. Microwave radiation can cause cell walls to rupture, forming small holes. The formation of holes allows cellulase to enter the cell walls. After enzymatic hydrolysis of cell walls, it is beneficial to the outflow of flavonoids from cells. Microwave-assisted enzymatic extraction has the advantages of saving time and reducing energy consumption. Moreover, compared with traditional extraction methods, microwave-assisted enzymatic extraction can improve the extraction efficiency of flavonoids to a certain extent. The schematic diagram of the microwave-assisted enzymatic extraction of flavonoids from *A. mume* Sieb. blossom is shown in Figure 1.

### 2.3. Determination of Flavonoid Content

#### 2.3.1. Establishing a Standard Curve

Sodium nitrite–aluminum nitrate colorimetry was used to construct a standard curve [17]. Dry rutin standard (50.0 mg) was weighed precisely and put into a 250 mL volumetric flask. The volumetric flask was filled with 250 mL of 75% ethanol. The standard solutions of 0.0, 1.0, 2.0, 3.0 L, 4.0, and 5.0 mL were individually pipetted and transferred into a 20 mL test tube. Sodium nitrite (5%, 2 mL) was added to each test tube then shaken well. The test tube was allowed to rest for 5 min, and then aluminum nitrate (10%, 2 mL) was added into the test tube. After the test tube rested for 5 min, 10 mL 4.3% sodium hydroxide solution was added. Then, the 75% ethanol was added into the test tube until the volume reached 20 mL. The concentration of rutin in these standardized samples was 0.0, 0.01, 0.02, 0.03, 0.04, and 0.05 mg/mL, respectively. The optical density (OD) was measured using a spectrophotometer (759S, Lengguang Co., LTD, Shanghai, China) at 510 nm after placing these standard samples at room temperature for 20 min. The standard curve was constructed using concentration (mg/mL) as abscissa and OD as ordinate.

#### 2.3.2. Measurement of Flavonoid Content

A 2 mL sample solution was drawn for each test. Then, the sample solution was added into a 20 mL volumetric flask, and other reagent solutions were added as detailed in the preceding section until the volume reached 20 mL. The OD was measured at 510 nm, as described in the preceding section. The flavonoid content was calculated using the standard curve. The flavonoid content (%) in the extract was calculated using Equation (1) [18].

Flavonoid content (100%) = C × N × V/M × 100(1)

where C represents the flavonoid content calculated using the calibration curve (mg/mL); N is the dilution factor; V is the total volume (mL) of the extract solution; M is the dry mass (mg) of *A. mume* Sieb. blossom.

### 2.4. Single Factor Test Design

Dried *A. mume* Sieb. blossom powder (10 g) was precisely weighed and put into a volumetric flask. Distilled water (200 mL) was added to the volumetric flask. Cellulase of different mass percentages (1.5%, 2.0%, and 2.5%), different microwave power (200, 250, and 300 W), different microwave treatment times (3, 4, and 5 min), and different enzymatic hydrolysis times (40, 50, and 60 min) was used for the single-factor experiment.

### 2.5. Orthogonal Experimental Design

Based on the results obtained from the single factor experimental design, cellulase, microwave power, microwave action time, and enzymatic hydrolysis time were considered. Each factor was designed to have three levels. The orthogonal test of L_9_(3^4^) was designed with the extraction of flavonoids as the index to optimize the process parameters of the microwave-assisted enzymatic extraction.

### 2.6. Mouse Treatment and Test Design

The mice were randomly divided into Dextran Sulphate Sodium (DSS) model, low-dose (FAMB-L), medium-dose (FAMB-M), high-dose (FAMB-H), and control groups, each having 12 mice. In addition to the control group, the mice in the other 4 groups were given water containing 5% DSS on day 1–6. On day 7–16, the mice of FAMB-L, FAMB-M, and FAMB-H groups were given 2 mL FAMB aqueous solution at different doses of 100, 150 and 200 mg/kg body weight, respectively. The mice of DSS model group were given 2 mL of DSS in drinking water. The mice of the control group were given 2 mL of distilled water daily.

### 2.7. Preparation of Mouse Spleen Lymphocyte Suspension

After the mice were sacrificed, their spleens were separated under aseptic conditions, and the fat and fascia of the spleen were removed. Then, the spleen was placed on a 200-mesh sieve, and the sieve was put into a glass petri dish. Phosphate buffer was poured into the petri dish, and the spleen was gently squeezed with a glass plate to separate mouse spleen lymphocytes. The cell mixture was centrifuged using a centrifuge at 955× *g* and 4 °C for 5 min. The supernatant was removed, and the red cell lysate was added. Then, the centrifuge tubes were rested for 5 min and centrifuged once again for 5 min. After cleaning twice with phosphate buffer, 5 mL RPMI-1640 medium was added to the centrifuge tube. Then, the cell concentration was adjusted to 2 × 10^5^ mL^−1^.

### 2.8. Measurements of Spleen and Thymus Indices

The mice were weighed and sacrificed. Spleens and thymuses of mice were collected and weighed. The spleen and thymus indices were calculated using Equation (2) [19].

Spleen or thymus index (mg/g) = spleen or thymus weight (mg)/mouse body weight (g). (2)

### 2.9. Measurements of Cytotoxicity of Natural Killer Cells (NK)

Mice spleen lymphocyte suspension was used as effector cells, and YAC-1 and P815 cells were used as target cells (4 × 10^4^ mL^−1^). The cells were seeded in a 96-well plate at 50:1 effector-to-target cell ratio. The cells were incubated for 5 h at 37 ℃ in a humidified incubator containing 5% CO_2_. Then, MTT solution (20 µL 5 mg/mL) was added and incubated for 4 h. Superstratum solution (100 µL) and DMSO solution (50 µL) were added to the pore of 96-well plate. After shaking, the OD of the solution was measured at 490 nm. At the same time, the ODs of the effector cell control, target cell control, and blank control were measured at 490 nm [20]. NK killing activity was calculated using Equation (3).

NK killing activity (%) = [target cell control OD value − (test cell OD value − effector cell control OD value)/target cell control OD value] × 100%.(3)

### 2.10. Measurements of TNF-α, IFN-γ, and IL-2 Concentrations in Serum

For these experiments, blood was collected from the eye sockets of the mice after they were sacrificed. The collected blood was placed into an Eppendorf tube. After standing at room temperature for 2 h, the samples were centrifuged at 2655× *g* for 10 min. The concentrations of serum cytokine (TNF-α, IFN-γ, and IL-2) were measured in the supernatant. The determination method was based on the instructions provided in the ELISA kits [21].

### 2.11. Measurements of Spleen Lymphocyte Apoptosis

The apoptotic cells of mice spleen lymphocyte suspension were determined by flow cytometry (BD FACS Aria II, NJ, USA) [22]. Spleen lymphocyte suspension of mice was centrifuged for 5 min at 424× *g*. The supernatant was then discarded. The precipitate was washed twice with PBS solution. The precipitated cell mass was then configured with PBS to obtain mice spleen lymphocyte suspension with a concentration of 2 × 10^5^ mL^−1^. Mice spleen lymphocyte suspension (100 µL), produced as described above, was added into the test tube. Then, 500 µL Binding Buffer, 5 µL Annexin V-FITC, and 5 µL Propidium Iodide (PI) were added into the test tube. The test tubes were mixed gently and placed in a dark place at room temperature for 15 min. PBS buffer solution (400 µL) was added to each test tube. The apoptotic status of each group was observed, and the apoptotic yield was calculated according to the flow two-dimensional diagram.

### 2.12. Statistical Analysis

SPSS 20.0 software (IBM, Chicago, IL, USA) was used to perform ANOVA. The significant difference between two mean values was determined by Duncan’s multiple comparison test. A 95% confidence level (*p* < 0.05) was used to confirm significant differences.

## 3. Results and Discussion

### 3.1. Calibration Curve of Rutin

**Figure 2:**
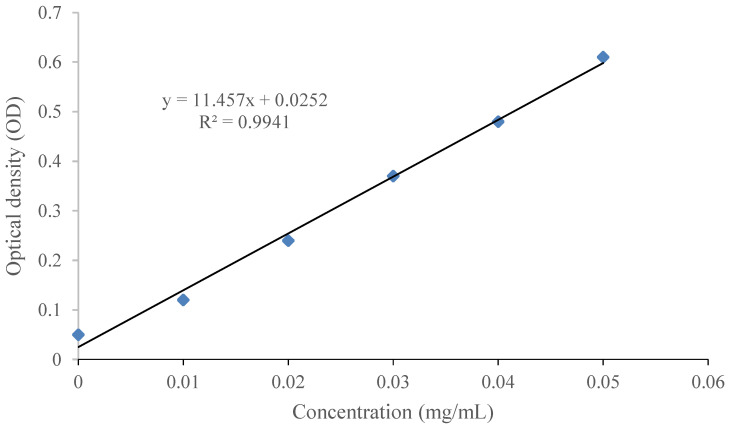
Standard calibration curve used to determine rutin concentration.

The calibration curve of rutin is shown in Figure 2. A linear equation (y (OD) = 11.457 × (concentration of rutin) + 0.0252) represented the data sets with R^2^ = 0.9941. This equation was used to determine the rutin concentration in test samples.

### 3.2. Single Factor Test Results

**Figure 3:**
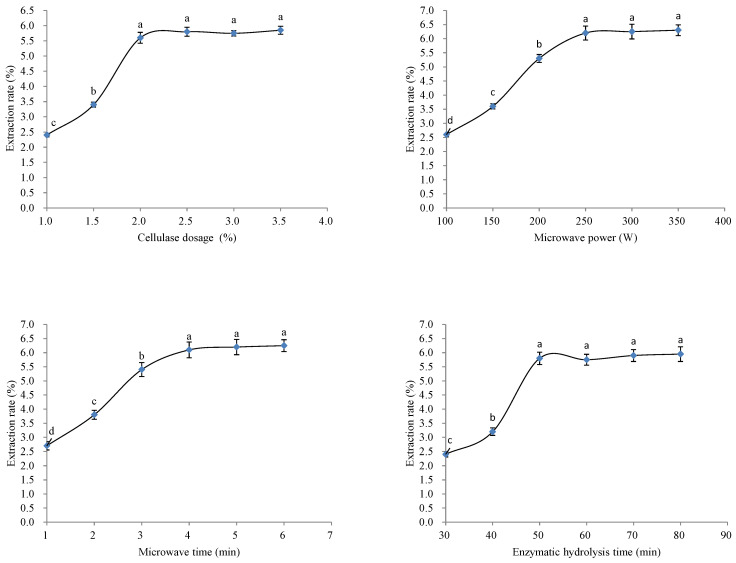
Effect of single factors on the flavonoid content in the extract. Different lowercase letters (a, b, c, d) in the same single factor express the significant differences (*p* < 0.05).

Figure 3 shows that with the increase in enzyme dosage, the flavonoid content in the extract of *A. mume* Sieb. blossom increased gradually. When the concentration of cellulase reached 2.0%, the flavonoid content in the extract was the highest. The flavonoid content in the extract did not increase when the enzyme dosage was increased above 2%. This indicated that the enzyme reached saturation level, and a further increase was unable to dissolve more flavonoids from *A. mume* Sieb. blossom. Similarly, when the microwave power was 200 W, the flavonoid content in the extract reached the peak level, and a further increase in microwave power did not significantly improve the extraction. When the microwave time was 4 min, the flavonoid content in the extract was the highest; however, when the microwave time was increased further, the flavonoid content in the extract did not increase significantly. The flavonoid content in the extract was the highest at the enzymatic hydrolysis time of 50 min and did not increase further when a longer hydrolysis time was used.

### 3.3. Orthogonal Test Results

Based on the results of the single factor experiments, cellulase dosage, microwave power, microwave time, and enzymatic hydrolysis time were taken as important factors, and three levels were designed for each factor (Table 1). Results of the orthogonal tests are shown in Table 2, and the analysis of variance is shown in Table 3.

As can be seen from Table 2, the degree of influence of cellulase dosage, microwave power, microwave time, and enzymatic hydrolysis time on the yield of flavonoids from *A. mume* Sieb. blossom was: cellulase dosage (A) > microwave time (C) > enzymatic hydrolysis time (D) > microwave power (B). Therefore, the optimal technological condition of the extraction of flavonoids from *A. mume* Sieb. blossom through microwave-assisted enzymatic extraction was A_2_B_2_C_3_D_2_. However, further analysis of variance is needed to determine whether or not the influence of these factors on the extraction of flavonoids was significant.

Table 3 shows the outcome of the analysis of variance of data obtained from the orthogonal test. The amount of cellulase, microwave time, and enzymatic hydrolysis time had a significant influence (*p* < 0.05) on the extraction yield of flavonoids, while microwave power had no significant influence on it. However, microwave power (B_1_) was also considered from the perspective of energy conservation. Therefore, the optimal combination of parameters in this microwave-assisted enzymatic extraction determined through the orthogonal experiment was A_2_B_1_C_3_D_2_, i.e., the cellulase dosage was 2.0%, the microwave power was 200 W, the microwave time was 5 min, and the enzymatic hydrolysis time was 50 min.

### 3.4. Effect of FAMB on Spleen and Thymus Indices

**Figure 4:**
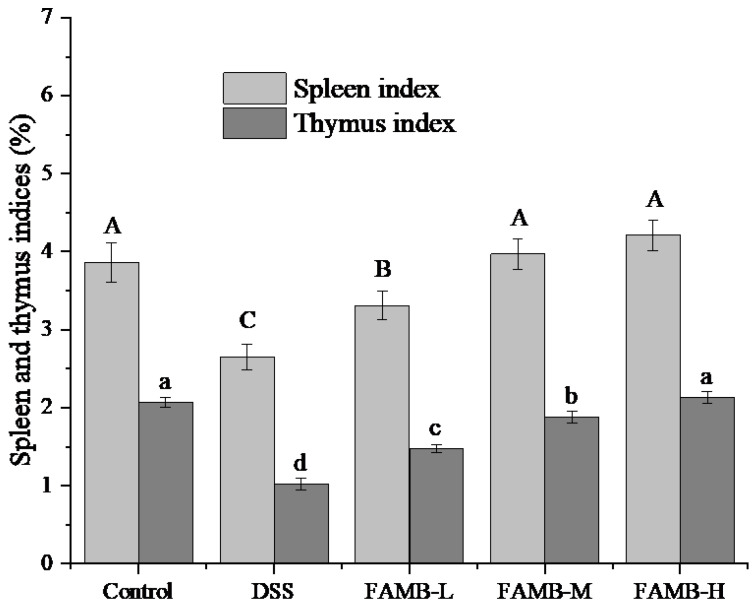
Effect of *A. mume* Sieb. blossom (FAMB) on spleen and thymus indices of colitis mice. Different uppercase and lowercase letters in the same index express the significant differences (*p* < 0.05). Uppercase (A, B, C) versus control groups for spleen index. Lowercase letters (a, b, c, d) versus control groups for thymus index.

The effect of FAMB on the spleen and thymus indices in DSS-induced colitis mice is shown in Figure 4. As can be observed, the spleen and thymus indices of the model group mice decreased significantly (*p* < 0.05), indicating the success of the model. After feeding FAMB, the spleen and thymus indices of the mice increased significantly (*p* < 0.05)) with the increase in the dose. These results showed that the feeding of the FAMB alleviated the injury of organs in mice.

The spleen and thymus are important indicators of innate immune function [23]. The spleen is among the important lymphatic organs which clears foreign body germs in the blood and produces immune material, such as immune globulin [24]. The thymus is a central immune organ which produces T lymphocytes. Therefore, the changes of spleen and thymus indices reflect the innate immune function [25]. As can be observed (Figure 4), FAMB significantly increased the weight of immune organs. These results showed that the intestinal disturbance caused by DSS resulted in a decrease in spleen and thymus indices in mice. When the FAMB was administered through feeding, the gastrointestinal digestion and absorption function of mice gradually recovered, and spleen and thymus indices also increased.

### 3.5. Effect of FAMB on NK Cytotoxicity

**Figure 5:**
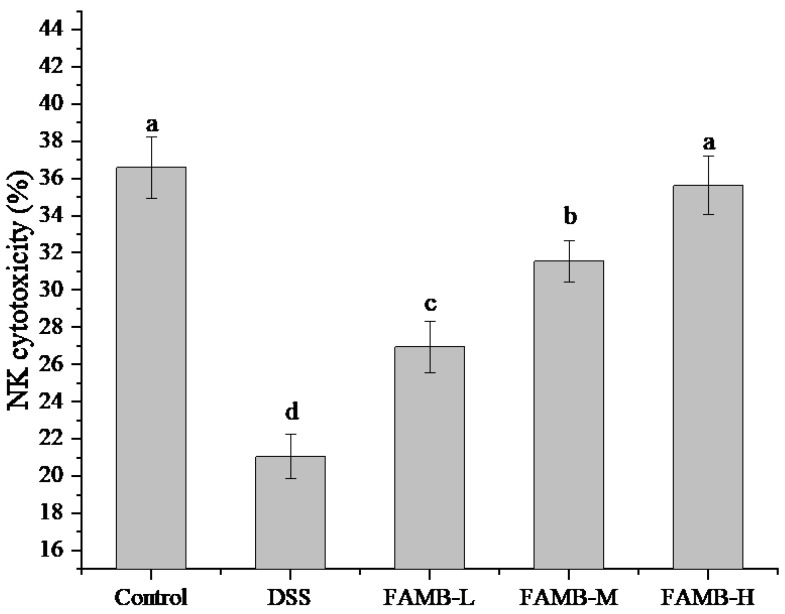
Effect of FAMB on natural killer (NK) cytotoxicity of colitis mice. Different lowercase letters (a, b, c, d) express the significant differences (*p* < 0.05).

The NK cytotoxicity of mice is shown in Figure 5. As can be observed, the NK cytotoxicity of the model group mice was significantly (*p* < 0.05) reduced, indicating that the model used in this work was successful. Compared with the model group, the NK cytotoxicity of the FAMB-L group was not significantly different. However, when a higher dose of FAMB was fed, the NK cytotoxicity in mice increased significantly (*p* < 0.05). The NK cytotoxicity of the FAMB-H group was not significantly (*p* > 0.05) different from that of the control group.

NK cells are important immune cells that not only regulate immune regulation but are also implicated in causing or promoting autoimmune diseases [26,27]. NK cells can directly kill infected cells and tumor cells without prior sensitization. Our data show that the NK cytotoxicity of colitis mice was significantly reduced, and the NK cytotoxicity was gradually restored after feeding FAMB, indicating that FAMB could boost the intestinal immune function of colitis mice.

### 3.6. Effect of FAMB on the Serum TNF-α, IFN-γ, and IL-2 Concentration

**Figure 6:**
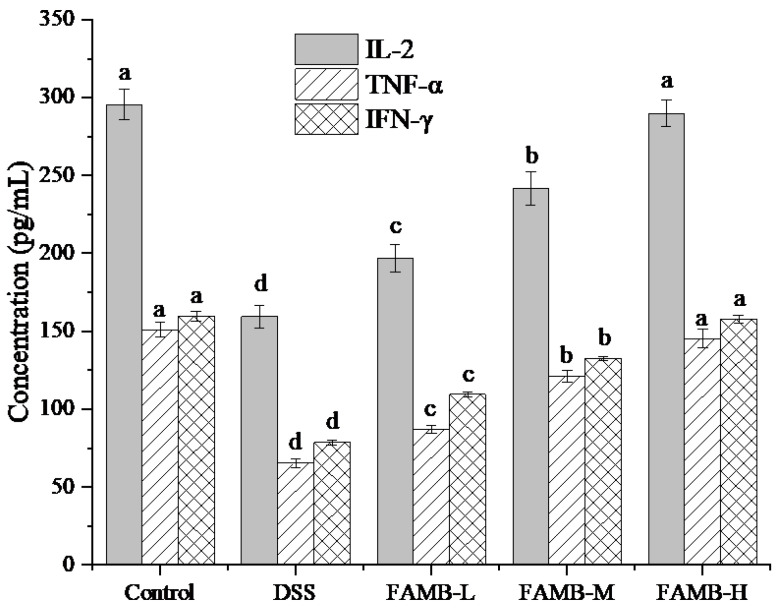
Effect of FAMB on the serum TNF-α, IFN-γ, and IL-2 concentrations of colitis mice. Different lowercase letters (a, b, c, d) in the same cytokine express the significant differences (*p* < 0.05).

The effect of FAMB on serum concentrations of TNF-α, IFN-γ, and IL-2 in mice is shown in Figure 6. As can be observed, the concentrations of TNF-α, IFN-γ, and IL-2 in the model group decreased significantly (*p* < 0.05), indicating that the model used here was realistic. In the FAMB group, the serum concentrations of TNF-α, IFN-γ, and IL-2 in mice were significantly increased (*p* < 0.05).

Cytokines are small molecular weight proteins secreted by immune cells and some non-immune cells and possess important biological activities [28]. Cytokines, as signal transduction molecules between cells, can stimulate hematopoietic function and regulate an immune response. TNF-α is important in host defense mechanisms and directly affects tumor cell death [29]. IFN-γ can also stimulate the expression of a range of inflammatory mediators and immune mediators. IFN-γ induces the expression of MHC molecules, inhibits viral replication, and activates macrophages [30]. IL-2 can participate in autoimmune reaction or inflammatory processes [31]. Our results show that the concentrations of TNF-α, IFN-γ, and IL-2 in serum were significantly reduced in DSS-induced colitis mice (*p* < 0.05). The concentrations of TNF-α, IFN-γ, and IL-2 in serum gradually increased after the mice were fed with FAMB and reached the same concentration as that in the high-dose and control groups. These results demonstrated that FAMB can regulate immune imbalance and help reduce the immunodeficiency.

### 3.7. Effect of on Spleen Lymphocyte Apoptosis

**Figure 7:**
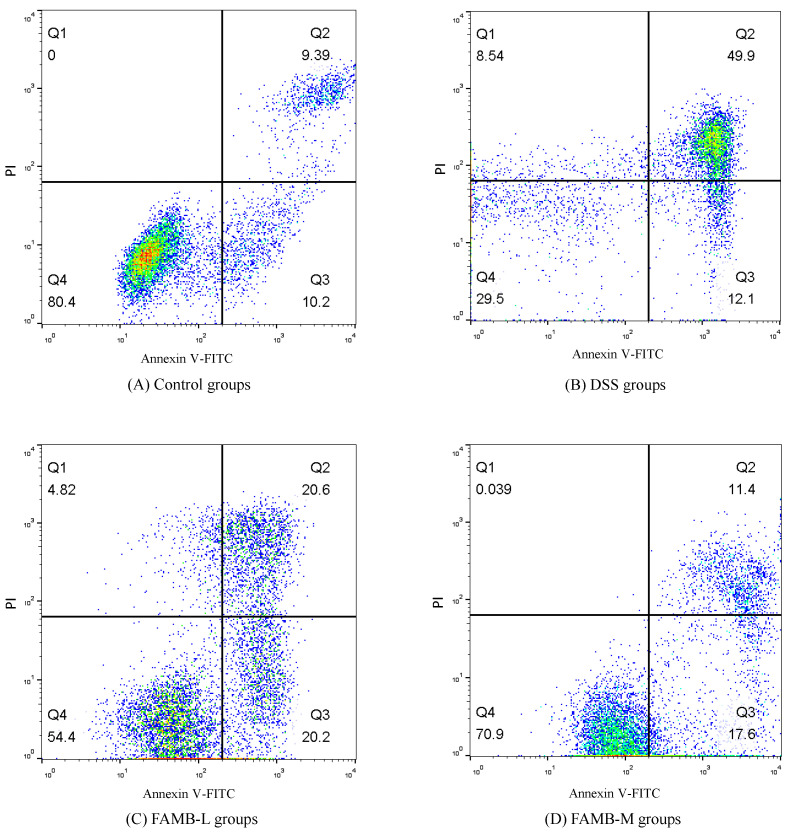

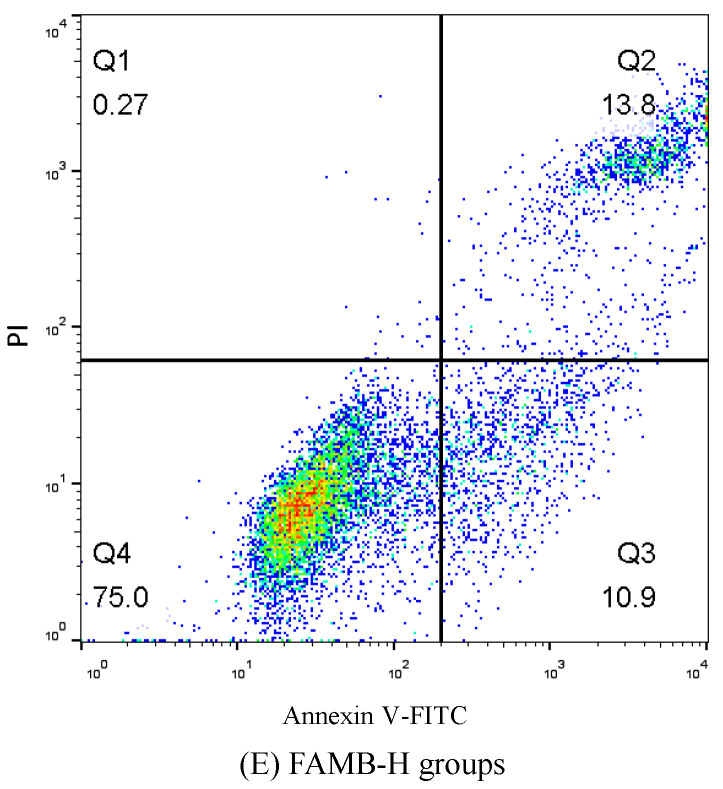
Effect of FAMB on the spleen lymphocyte apoptosis of colitis mice.

Table 4 and Figure 7 show the effects of FAMB on the apoptosis of spleen lymphocytes in colitis mice. These results showed that the apoptosis rate of spleen lymphocytes in the model group was three times as high as that in the control group. In the FAMB group, the level of apoptosis of spleen lymphocytes decreased gradually with the increase in FAMB dose. Apoptosis is a response of cells to environmental stimulus signals [32]. It is also considered to be a programmed mode of cell death [33]. Our results show that the DSS-induced apoptosis of spleen lymphocytes in colitis mice was significantly increased (*p* < 0.05). After the feeding the mice with FAMB, the degree of apoptosis of spleen lymphocyte decreased gradually and decreased to 24.70% in the high-dose group. These results demonstrated that FAMB could restore DSS-induced apoptosis of spleen lymphocytes in colitis mice.

## 4. Conclusions

The efficacy of the microwave-assisted enzymatic extraction of flavonoids from *A. mume* Sieb. blossom (FAMB) and the immunomodulatory effects of extracted flavonoids on mice with colitis were studied. The yield or efficacy of the extraction of FAMB using this method was affected by four parameters in the following order: the amount of cellulase > microwave treatment time > enzymatic hydrolysis time > microwave power. The variation of cellulase, microwave treatment time, and enzymatic hydrolysis time had a significant influence on the yield or efficacy of flavonoids, while the variation of microwave power had no significant influence. From the perspective of energy saving and efficacy of extraction, the cellulase dosage of 2.0%, the microwave power of 200 W, the microwave time of 5 min, and the enzymatic hydrolysis time of 50 min were found to be the optimum parameters.

DSS-induced colitis mice showed significant decreases in spleen and thymus indices; NK cytotoxicity; and TNF-α, IFN-γ, and IL-2 concentrations in serum. When these mice were fed with FAMB, the spleen and thymus indices increased significantly; NK cytotoxicity and TNF-α, IFN-γ, and IL-2 concentrations also increased in the serum. The degree of spleen lymphocyte apoptosis in colitis mice gradually decreased with the increase in FAMB feeding dose. These results showed that the FAMB can improve the body’s immune function and minimize the symptoms associated with low immunity.

## Figures and Tables

**Table 1:** Factors and levels of factors used in orthogonal test of microwave-assisted enzymatic extraction.

Levels	Factors			
	Cellulase Dosage A (%)	Microwave Power B (W)	Microwave Time C (min)	Enzymatic Hydrolysis Time D (min)
1	1.5	200	3	40
2	2.0	250	4	50
3	2.5	300	5	60

**Table 2:** The result of orthogonal test.

Serial Number	Cellulase Dosage A (%)	Microwave Power B (W)	Microwave Time C (min)	Enzymatic Hydrolysis Time D (min)	Flavonoid Content in Extract (%)
1	1.5	200	3	40	5.85 ± 0.13 ^c^
2	1.5	250	4	50	6.45 ± 0.16 ^a^
3	1.5	300	5	60	6.55 ± 0.21 ^a^
4	2.0	200	4	60	6.25 ± 0.11 ^a,b^
5	2.0	250	5	40	6.35 ± 0.19 ^a,b^
6	2.0	300	3	50	6.20 ± 0.15 ^a,b^
7	2.5	200	5	50	6.15 ± 0.12 ^b^
8	2.5	250	3	60	5.91 ± 0.09 ^c^
9	2.5	300	4	40	5.59 ± 0.05 ^d^
K_1_	18.850	18.250	17.960	17.790	
K_2_	18.800	18.710	18.290	18.800	
K_3_	17.650	18.340	19.050	18.710	
k_1_	6.283	6.083	5.987	5.930	
k_2_	6.267	6.237	6.097	6.267	
k_3_	5.883	6.113	6.350	6.237	
R	0.400	0.154	0.363	0.337	

Results are mean ± standard deviation. Different lowercase letters in the same column within the same sample express the significant differences (*p* < 0.05). K is the sum of flavonoid content at each level of each factor, and k is the average of each level. R is the range analysis, i.e., the maximum of the k minus the minimum of the k.

**Table 3:** The result of the analysis of variance of the data obtained from orthogonal test.

Variance Source	Squared Sum	Degrees Freedom	Mean Square	F-Value	Significance
A	0.307	2	0.154	7.754	*
B	0.040	2	0.020	1.000	
C	0.208	2	0.104	5.257	*
D	0.208	2	0.104	5.257	*
Error	0.040	2	0.020		

Note: F_0.01 (2,2)_ = 4.00; F_0.05 (2,2)_ = 9.00; * expresses the significant differences (*p* < 0.05).

**Table 4:** Effect of FAMB on the spleen lymphocyte apoptosis of colitis mice.

Groups	Numbers of Mice	Apoptosis Rate (%)
Control	12	19.59 ± 0.76 ^e^
DSS	12	62.00 ± 1.58 ^a^
FAMB-L	12	40.80 ± 1.05 ^b^
FAMB-M	12	29.00 ± 0.62 ^c^
FAMB-H	12	24.70 ± 0.66 ^d^

Results are mean ± standard deviation. Different lowercase letters in the same column within the same sample express the significant differences (*p* < 0.05).

## Data Availability

The data generated during the present study are available from the corresponding author on reasonable request.

## References

[1] Shi J., Gong J., Liu J., Wu X., Zhang Y. (2009). Antioxidant Capacity of Extract from Edible Flowers of A. mume Sieb. in China and Its Active Components. LWT-Food Sci. Technol..

[2] Nakamura S., Fujimoto K., Matsumoto T., Nakashima S., Ohta T., Ogawa K., Matsuda H., Yoshikawa M. (2013). Acylated sucroses and acylated quinic acids analogs from the flower buds of Prunus mume and their inhibitory effect on melanogenesis. Phytochemistry.

[3] Fujimoto K., Nakamura S., Matsumoto T., Ohta T., Ogawa K., Tamura H., Matsuda H., Yoshikawa M. (2013). Medicinal Flowers. XXXVIII. Structures of Acylated Sucroses and Inhibitory Effects of Constituents on Aldose Reducatase from the Flower Buds of Prunus mume. Chem. Pharm. Bull..

[4] Nakamura S., Fujimoto K., Matsumoto T., Ohta T., Ogawa K., Tamura H., Matsuda H., Yoshikawa M. (2013). Structures of acylated sucroses and an acylated flavonol glycoside and inhibitory effects of constituents on aldose reductase from the flower buds of Prunus mume. J. Nat. Med..

[5] Lei H., Ren R., Sun Y., Zhang K., Zhao X., Ablat N., Pu X. (2020). Neuroprotective Effects of Safflower Flavonoid Extract in 6-Hydroxydopamine-Induced Model of Parkinson’s Disease May Be Related to its Anti-Inflammatory Action. Molecules.

[6] Guo C., Shan Y., Yang Z., Zhang L., Ling W., Liang Y., Ouyang Z., Zhong B., Zhang J. (2020). Chemical composition, antioxidant, antibacterial, and tyrosinase inhibition activity of extracts from Newhall navel orange (Citrus sinensis Osbeck cv. Newhall) peel. J. Sci. Food Agric..

[7] Sae-Tan S., Kumrungsee T., Yanaka N. (2020). Mungbean seed coat water extract inhibits inflammation in LPS-induced acute liver injury mice and LPS-stimulated RAW 246.7 macrophages via the inhibition of TAK1/IκBα/NF-κB. J. Food Sci. Technol..

[8] Ren F., Nian Y., Perussello C.A. (2020). Effect of storage, food processing and novel extraction technologies on onions flavonoid content: A review. Food Res. Int..

[9] Rocchetti G., Pagnossa J.P., Blasi F., Cossignani L., Piccoli R.H., Zengin G., Montesano D., Cocconcelli P.S., Lucini L. (2020). Phenolic profiling and in vitro bioactivity of Moringa oleifera leaves as affected by different extraction solvents. Food Res. Int..

[10] Mai X., Liu Y., Tang X., Wang L., Lin Y., Zeng H., Luo L., Fan H., Li P. (2020). Sequential extraction and enrichment of flavonoids from Euonymus alatus by ultrasonic-assisted polyethylene glycol-based extraction coupled to temperature-induced cloud point extraction. Ultrason. Sonochem..

[11] Zhang L., Xu Q., Li L., Lin L., Yu J., Zhu J., Zhang H., Xia G., Zang H. (2020). Antioxidant and enzyme-inhibitory activity of extracts from Erigeron annuus flower. Ind. Crop. Prod..

[12] Zhang Z., Poojary M.M., Choudhary A., Rai D.K., Tiwari B.K.B.K. (2018). Comparison of selected clean and green extraction technologies for biomolecules from apple pomace. Electrophoresis.

[13] Arias J., Mejía J., Córdoba Y., Martínez J.R., Stashenko E.E., Del Valle J.M. (2020). Optimization of flavonoids extraction from Lippia graveolens and Lippia origanoides chemotypes with ethanol-modified supercritical CO_2_ after steam distillation. Ind. Crop. Prod..

[14] Moučková K., Pacheco-Fernández I., Ayala J.H., Bajerová P., Pino V. (2020). Evaluation of Structurally Different Ionic Liquid-Based Surfactants in a Green Microwave-Assisted Extraction for the Flavonoids Profile Determination of *Mangifera* sp. and *Passiflora* sp. Leaves from Canary Islands. Molecules.

[15] Ke L., Chen H. (2016). Enzymatic-Assisted Microwave Extraction of Total Flavonoids from Bud of *Chrysanthemum indicum* L. and Evaluation of Biological Activities. Int. J. Food Eng..

[16] Wen P., Hu T.-G., Linhardt R.J., Liao S., Wu H., Zou Y. (2019). Mulberry: A review of bioactive compounds and advanced processing technology. Trends Food Sci. Technol..

[17] Wang X., Wu Q.-N., Niu Q. (2017). Trace Elements Characteristic Based on ICP-AES and the Correlation of Flavonoids from Sparganii rhizoma. Biol. Trace Element Res..

[18] Chen H., Wang J., Liu X., Zhou A., Xiao J., Huang K., Chen H., Cao Y. (2020). Optimization in continuous phase-transition extraction of crude flavonoids from finger citron fruit and evaluation on their antiaging activities. Food Sci. Nutr..

[19] Fu Z., Wei Z., Miao M. (2018). Effects of total flavonoids of raspberry on perimenopausal model in mice. Saudi J. Biol. Sci..

[20] Hou D., Wang D., Ma X., Chen W., Guo S., Guan H. (2017). Effects of total flavonoids of sea buckthorn (*Hippophae rhamnoides* L.) on cytotoxicity of NK92-MI cells. Int. J. Immunopathol. Pharmacol..

[21] Song X., Xiao H.-T., Liao C.-H., Li L., Kang Q.-R., Jiang Y.-C., Hu X.-P., Zheng K., Fan L., He Z. (2017). Natural Products: The Master Regulators of Antiviral Cytokines. Curr. Org. Chem..

[22] Ishfaq M., Chen C., Bao J., Zhang W., Wu Z., Wang J., Liu Y., Tian E., Hamid S., Li R. (2019). Baicalin Ameliorates Oxidative Stress and Apoptosis by Restoring Mitochondrial Dynamics in the Spleen of Chickens Via the Opposite Modulation of NF-Kappab and Nrf2/HO-1 Signaling Pathway During Mycoplasma Gallisepticum Infection. Poult Sci..

[23] Liu X.Y., Xu L., Wang Y., Li J.X., Zhang Y., Zhang C., Wang S.S., Zhang X.M. (2017). Protective Effects of Total Flavonoids of Astragalus Against Adjuvant-Induced Arthritis in Rats By Regulating OPG/RANKL/NF-Kappab Pathway. Int. Immunopharmacol..

[24] Fu Y.-F., Jiang L.-H., Zhao W.-D., Xi-Nan M., Huang S.-Q., Yang J., Hu T.-J., Chen H. (2017). Immunomodulatory and antioxidant effects of total flavonoids of Spatholobus suberectus Dunn on PCV2 infected mice. Sci. Rep..

[25] Ince E. (2020). The protective effect of quercetin in the alcohol-induced liver and lymphoid tissue injuries in newborns. Mol. Biol. Rep..

[26] Sassi A., Maatouk M., El Gueder D., Bzéouich I.M., Hatira S.A.-B., Jemni-Yacoub S., Ghedira K., Chekir-Ghedira L. (2018). Chrysin, a natural and biologically active flavonoid suppresses tumor growth of mouse B16F10 melanoma cells: In vitro and In vivo study. Chem. Interactions.

[27] Han R., Wu W.-Q., Wu X.-P., Liu C.-Y. (2015). Effect of total flavonoids from the seeds of Astragali complanati on natural killer cell function. J. Ethnopharmacol..

[28] Zhu K., Huang G., Xie J., Zhou X., Mu J., Zhao X. (2019). Preventive effect of flavonoids from Wushan Shencha ( Malus doumeri leaves) on CCl 4 -induced liver injury. Food Sci. Nutr..

[29] Zhang X.-X., Wu Q., Yan Y.-L., Zhang F.-L. (2017). Inhibitory effects and related molecular mechanisms of total flavonoids in Mosla chinensis Maxim against H1N1 influenza virus. Inflamm. Res..

[30] Yang J.X., Maria T.C., Zhou B., Xiao F.L., Wang M., Mao Y.J., Li Y. (2020). Quercetin Improves Immune Function in Arbor Acre Broilers Through Activation of NF-Kappab Signaling Pathway. Poult Sci..

[31] Dey P., Chaudhuri T.K. (2014). In vitro modulation of TH1 and TH2 cytokine expression by edible tuber of Dioscorea alata and study of correlation patterns of the cytokine expression. Food Sci. Hum. Wellness.

[32] Cerqueira F., Cordeiro-Da-Silva A., Araújo N., Cidade H., Kijjoa A., Nascimento M.S.J. (2003). Inhibition of Lymphocyte Proliferation by Prenylated Flavones: Artelastin Aas A Potent Inhibitor. Life Sci..

[33] Yang X., Sun Y., Xu Q., Guo Z. (2006). Synthesis and immunosuppressive activity of l-rhamnopyranosyl flavonoids. Org. Biomol. Chem..

